# A Demonstration of the Transition from Ready-to-Hand to Unready-to-Hand

**DOI:** 10.1371/journal.pone.0009433

**Published:** 2010-03-09

**Authors:** Dobromir G. Dotov, Lin Nie, Anthony Chemero

**Affiliations:** 1 Center for the Ecological Study of Perception and Action, University of Connecticut, Storrs, Connecticut, United States of America; 2 Scientific and Philosophical Studies of Mind Program, Franklin & Marshall College, Lancaster, Pennsylvania, United States of America; Mount Sinai School of Medicine, United States of America

## Abstract

The ideas of continental philosopher Martin Heidegger have been influential in cognitive science and artificial intelligence, despite the fact that there has been no effort to analyze these ideas empirically. The experiments reported here are designed to lend empirical support to Heidegger's phenomenology and more specifically his description of the transition between ready-to-hand and unready-to-hand modes in interactions with tools. In experiment 1, we found that a smoothly coping cognitive system exhibits 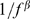 type positively correlated noise and that its correlated character is reduced when the system is perturbed. This indicates that the participant and tool constitute a self-assembled, extended device during smooth coping and this device is disrupted by the perturbation. In experiment 2, we examine the re-organization of awareness that occurs when a smoothly coping, self-assembled, extended cognitive system is perturbed. We found that the disruption is accompanied by a change in attention which interferes with participants' performance on a simultaneous cognitive task. Together these experiments show that a smoothly coping participant-tool system can be temporarily disrupted and that this disruption causes a change in the participant's awareness. Since these two events follow as predictions from Heidegger's work, our study offers evidence for the hypothesized transition from readiness-to-hand to unreadiness-to-hand.

## Introduction

Ever since the influential critiques by Hubert Dreyfus [1], [2] researchers in AI and cognitive science have been interested in, or at least aware of, the work of phenomenological philosopher Martin Heidegger. Initially, Dreyfus argued that Heidegger's insights into the nature of human thinking and experience made artificial intelligence impossible. More recently, it has been argued that Heidegger's insights into the nature of thinking and experience lead to a different approach to creating artificial intelligence and studying cognition scientifically [3]–[8]. Despite widespread attention in cognitive science and artificial intelligence to Heidegger's work, this interest has remained largely conceptual and no effort has been made to put Heidegger's theory in an experimental framework. A search of the PsycINFO database on December 10, 2009, found no articles concerning Heidegger that involved laboratory work. The reasons behind this lack of an experimental approach are easy to understand once one acknowledges that phenomenology is not a psychological discipline and there is no established correspondence between the two; one cannot directly test Heidegger's concepts using the tools of psychological science because his is not a psychological theory. Yet, the current paper makes a first attempt to fill this gap by deriving testable predictions that follow from specific aspects of the philosopher's work. The use of phenomenological studies to inform experimental design, an approach called *front-loading phenomenology*, has been proposed as one way to naturalize phenomenology [9].

The portion of Heidegger's philosophical thinking that has been considered relevant to cognitive science is found in Division I of his great early work *Being and Time*. In this paper, we follow the “analytic” interpretation of Heidegger's work (e.g., [10]). This is appropriate, whether or not it is the best way to understand Heidegger, because the analytic reading is the only one that has had any influence in the cognitive sciences. In Chapter III of Being and Time, Heidegger distinguishes three modes of experiencing the world. Most human activity, Heidegger argued, is absorbed, skillful engagement with entities in the world. When we are coping skillfully with the world, we experience entities around us as ready-to-hand. To use Heidegger's example, a hammer is encountered ready-to-hand, as a piece of equipment, when it is being simply used to drive in nails. Our engagement with entities ready-to-hand does not involve explicit awareness of their properties; instead, we “see through” them to the task we are engaged in. When we are smoothly driving in nails with a hammer, our focus is on the thing we are building not the size or shape or color of the hammer.

Heidegger argues that skilled coping, when we engage with entities as ready-to-hand, is our primary way of engaging with the world. Sometimes, though, our skillful coping is temporarily disturbed. When this happens, we encounter entities as unready-to-hand. When we go from smoothly hammering to having difficulty, our experience of the previously ready-to-hand entities changes: we experience the hammer, nails and board as failing to serve their function appropriately. The hammer is too light or heavy, the nails are too soft, the board has an unfortunately placed knot. When we encounter entities as unready-to-hand, we experience them as frustrating our coping with the world, and we must focus closely on our activity. As Blattner [11] puts it, unreadiness-to-hand is a deficient mode of readiness-to-hand. We are still using the piece of hardware to complete a task, but our experience of the situation has changed. We can no longer “see through” the tool to focus on the task; instead, we must explicitly attend to the unready-to-hand object that the tool has turned into.

Heidegger's third way of experiencing the world is as present-at-hand. The hammer is encountered as present-at-hand when we stop hammering and consider the hammer's shape or color or weight; when considered this way the hammer is no longer a useful tool but merely an object with various properties. Heidegger argued that readiness-to-hand is primary in two ways. First, the majority of our experience of the world is engaging with entities ready-to-hand. Second, readiness-to-hand is, from a phenomenological standpoint, ontologically primary while the other modes are derivative of it. The true nature of the tools we experience is their way of being ready-to-hand. Thus, a hammer is primarily something used in building, and only secondarily something we are temporarily having trouble using or something with a particular shape, color and chemical composition.

Even these very brief remarks are enough to make sense of what many have taken to be the import of Heidegger's phenomenology for the cognitive sciences. When you are smoothly coping with a hammer that is ready-to-hand, the ready-to-hand hammer recedes in your experience, and your focus is on the task you are completing. Your experience of the hammer is no different than the experience of the hand with which you are wielding it. This has inspired the hypothesis of extended cognition, i.e., the claim that cognitive systems sometimes extend beyond the biological body [6], [12]–[16]. Hammers and other tools that are ready-to-hand are literally part of the cognitive system. When a tool malfunctions, however, and becomes unready-to-hand, it becomes the object of primary concern; it is no longer part of the extended cognitive system, rather it is the thing that that the cognitive system is concerned with.

These remarks also point to Dreyfus's critique of artificial intelligence, at least as it was practiced in the 1960s and 1970s in what Haugeland calls Good Old Fashioned AI (GOFAI). In GOFAI, a system's knowledge of the environment is represented as a series of logical propositions. Thus, a GOFAI system's representation of a hammer might include the following: it is a 1-inch diameter cylinder, with a complex shape attached to one end and a rubber coating on the other; it weighs 22 ounces; the center of inertia is very close to the end with the complex shape; it is made of steel; portions of it are painted blue, other portions are unpainted; it can be used to hammer nails. But if Heidegger is correct about our modes of engagement with the world, humans are typically unaware of the weight, shape, color and center of inertia of hammers. Instead they encounter them as ready-to-hand, while using them to hammer nails. In fact, most of the time when humans encounter hammers, they are not explicitly aware of any of their properties: they use them skillfully, seeing through them to the task at hand. Knowledge representation in GOFAI is, perhaps, sufficient to capture the way humans experience things as present-at-hand. But this is not the way humans typically experience things, nor is it the way things most fundamentally are. Thus, Dreyfus argued, AI (circa 1960s and 1970s) would never succeed, nor would any scientific psychology that shared its assumptions.

Things have changed dramatically in AI and cognitive science since the time Dreyfus made these arguments, and at least some of the changes have been made explicitly in response to Dreyfus's critiques. But notice that the Dreyfus arguments depend on the correctness of Heidegger's phenomenological analysis. The credibility of Heidegger's analysis of our ways of engaging with the world has, thus far, depended on something like its face validity: think about your experience, does it match up with what Heidegger describes? Of course, not everyone gives the same answer to this question.

**Figure 1:**
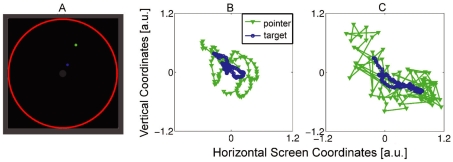
The visual playground environment. A single frame (a) captured during the course of a trial is shown and visible inside it are the pen, the gray center, and blue and green dots for the target and pointer objects, respectively. Representative pointer and target object trajectories on the screen from three-second excerpts with a normally behaving (b) and impaired (c) mouse are portrayed.

The two experiments described below provide the beginning of an empirical basis for Heidegger's phenomenology: in particular, they demonstrate the correctness of his hypothesized transition from readiness-to-hand to unreadiness-to-hand. In order to investigate this transition, we constructed in the laboratory a situation involving equipment that participants are already familiar with. The participant plays a simple video game, using a computer mouse directly linked to a pointer figure on a computer screen to steer a target figure into a designated area (See Figure 1a). The task resembles a video game version of herding, as when a dog keeps a sheep inside a proscribed area. What allows the participant to guide the target is that it always tries to escape away from the pointer in a semi-predictable fashion. To make an analogy to Heidegger's example, here the mouse plays the role of the handle and the on-screen pointer figure plays a role similar to that of the hammer striking face. About thirty seconds from the beginning of the trial a perturbation in the mapping between mouse movement and pointer movement instantiates equipment malfunctioning. It lasts a few seconds and then the situation returns to normal.

Heidegger's phenomenological philosophy would predict that prior to the perturbation, the participant in the experiment will smoothly cope with the tool as a ready-to-hand tool; during the perturbation, the mouse will become unready-to-hand and the participant will be forced to focus her attention and cognitive resources on the mouse and mouse-pointer coupling and, by necessity, away from a secondary cognitive task. In the first experiment, we use relatively novel tools to analyze the dynamical structure in the hand-mouse movements to examine at the level of motor control the nature of skillful coping and its breakdown induced by the temporary disturbance in mouse behavior. In the second experiment, we show that this disturbance causes an attentional shift and a temporary re-allocation of cognitive resources. For the sake of consistency, the two experiments used the same experimental design, that is, both experiments involved a herding motor task and a verbalized counting cognitive task with only slight differences in some parameters.

Ideally, we would be able to show explicitly that attention is shifted towards the misbehaving tool as it has lost its transparency. In the current study we are restricted only to an implicit test, namely, attention to a second task is reduced, which is what the ready-to-hand to unready-to-hand transition in conjunction with a contemporary understanding of attentional resources predicts.

### Experiment 1

Using motion-tracking equipment we recorded the three-dimensional trajectory of the hand-tool system (from now on called the hand-tool for simplicity). Research on the generation of purposeful motor behavior in comparable tasks demonstrates that the variability of the movement time series has an intrinsic role in the generation of the movements, and is not mere additive noise “blurring” the signals coming from the central nervous system (for a review, see [17] or, for a more analytical critique of the conception of noise and signal in physiology and the life sciences in general, [18]). In order to characterize the fluctuations in the movements of the hand-tool before, during, and after the perturbation, we applied an analysis that has been used to establish long-range correlations in the time series which are expressed as 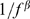 type noise in the frequency domain [19]. The significance of 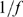 noise for behavioral data and for our experiment needs special attention.

The technical meaning of classifying variability in human behavior as 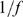 noise [20] is that activity magnitude scales across the frequency spectrum invariantly, that is, as a power-law with unity exponent 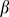 in 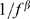. Usually the discussion of 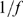 noise in behavioral time series includes the more general class of 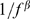 type noise without positing a categorical difference between the two as long as 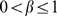 [21], [22]. Data where 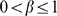 are also said to be a long-range correlated series or have long memory, and this notion of long memory has become the epitome of scale-invariant behavior [23], [24]. Testing for the presence of a power-law long memory process at the motor control level is the main goal of the first experiment. Such power-law scaling has been associated with a sort of extended cognitive systems that will be argued to follow from Heidegger's phenomenological philosophy. van Orden *et al.* [21] and Holden *et al.* [25] argue that 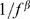 noise found in an inventory of cognitive tasks is a signature of a softly assembled system exhibiting and sustained by *interaction-dominant dynamics*, and not *component-dominant dynamics*. In component-dominant dynamics, behavior is the product of a rigidly delineated architecture of modules, each with pre-determined functions; in interaction-dominant dynamics, on the other hand, coordinated processes alter one another's dynamics, with complex interactions extending to the body's periphery and, sometimes, beyond. When, as part of an experiment, a participant is repeating a word, a portion of her bodily and neural resources, along with environmental support structures, assemble themselves into a “word-naming device” [21, p.346]. Device assembly as the product of interactions within and across the temporal and spatial scales of elemental activity can account for the 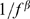 character of behavioral data, while assembly by virtue of rigid components with predetermined roles and fixed communication channels cannot [26]. Thus we can take the presence of a 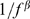 long memory process as indicative of the activity of a smoothly operating system, softly assembled by virtue of interaction-dominant dynamics. For the sake of brevity, we use the initials “IDS” to designate such systems.

By looking for 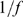 noise recorded at the interface of body and tool, we address the hypothesis that, while smoothly operating an instrument, a human performer instantiates such an IDS spanning the extended body-tool system. The first of the predictions from Heidegger's phenomenological philosophy that we laid out in the introduction can now be expressed in a more operational form.

1. When participants are smoothly operating the tool to play the computer game, i.e., when the participants are interacting with the tool as ready-to-hand, it becomes part of the IDS solving the problem posed by the game. Given this, we predict that the variability recorded at the body-tool interface is 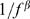 type noise or a long memory process.
2. When, during the experiment, the connection between mouse movements and pointer movements on the screen is perturbed, the participants should experience a breakdown in their smooth coping and, consequently, experience the tool as unready-to-hand. When this occurs, we predict that the variability recorded at the body-tool interface will not have 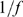 structure, showing a disruption in the IDS.

The experiment involved an additional cognitive task of counting backwards by three out loud. This task was only used to keep the conditions consistent across experiments, however, and performance in it was not assessed.

### Experiment 2

Even if the results of experiment 1 successfully reveal that the hand-tool, as a part of a larger smoothly-coping IDS, becomes functionally removed from it during the perturbation, this would only go part of the way to demonstrating Heidegger's proposed transition from readiness-to-hand to unreadiness-to-hand. Heidegger's distinction also implies a change in experience: since we can no longer see through the unready-to-hand entity to the task we are using it to complete, it will attract our attention and consequently impair performance in a secondary task. For this reason experiment 2 employed a divided attention paradigm and focused on the secondary cognitive task of counting out loud. Put in terms of experiment 1, the participant shifts from attending to the counting and the “herding” tasks to needing to attend to the counting, herding, and tool handling tasks. To validate this emergence of an additional object of attention we interpret the task of experiment 1 within the light of longstanding results in cognitive psychology concerning attention and cognitive abilities [27], [28]. Attention is a limited resource; changing the way that attention is allocated among multiple tasks affects cognitive performance [29]–[31]. This suggests that the redistribution of attentional resources accompanying a shift from readiness-to-hand to unreadiness-to-hand should have an impact on the performance of a demanding cognitive task. This leads to the prediction that counting rate with mouse perturbation will decrease, as participants are able to allocate less attention to that task. Consequently, our study can support the validity of the hypothesized transition by way of finding data that conform to a theory-derived prediction.

## Materials and Methods

### Participants and Procedure

Experiment 1 was approved by the University of Connecticut Institutional Review Board. Experiment 2 was approved by the Franklin & Marshall College Institutional Review Board. All participants in both experiments signed informed consent forms.

Undergraduate students (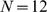) at University of Connecticut participated in the first experiment for credit. At the beginning, the experimenter explained that the nature of the task was to investigate motor control in the context of playing a computer game while also performing an additional cognitive task: verbally counting backwards by three. In regard to the main task, the specific instructions given were to, first, try to keep the target figure within or as close as possible to the gray circle in the middle of the “pen” area and, second, not let the target leave the screen. An example of performing both tasks was given and then the participant was allowed a few practice trials with no mouse perturbation. The goal of the practice trials was to make sure the participant could maintain control over the target object on the screen while also counting. Once the participant felt comfortable with both tasks, six experimental trials were performed. Because participants were allowed practice trials and conditions were exactly the same in every trial, there was no need for more than six trials. Furthermore, we did not want practice effects to interfere with our results as participants began to expect the perturbation.

Experiment 2 took place at Franklin and Marshall College and used the same design as experiment 1 with a few small modifications. All participants (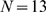) were given a similar set of instructions as in experiment 1, plus that they would be videotaped during the experiment. After they became comfortable with the dot herding task during practice trials with no perturbation, they were introduced to the additional counting task, i.e. counting backwards by three. Only a single experimental trial was necessary because the analysis of cognitive performance was much more straightforward than the motor behavior one and the effect of the perturbation was quite telling.

### Apparatus

The participants sat in front of a table with a computer, monitor, and a mouse. A custom Matlab (Mathworks, Natick, MA) script was used for a 60-second-long visuomotor coordination task involving the mouse and monitor. We took advantage of the PsychoPhysics Toolbox [32]–[34] the main purpose of which is to give our script nearly real-time control over the computer operating system kernel. Before each step the script records current mouse position and prepares a frame with pointer and target locations. A frame is sent to the screen for update every 35 milliseconds. The result is a seemingly real-time relation between mouse and pointer and between pointer and target.

Before a trial starts, a target blue dot and a mouse-controlled green pointer dot stay in the center of a playground area delineated by a red ellipse - the pen - on the computer screen. Beginning with the start of the trial, the pointer is released and the target starts being displaced away from the pointer by a vector defined by the following equation
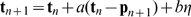
(1)where 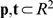 are vectors of the computer screen Cartesian coordinates for the pointer and target objects, respectively, 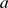 and 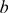 are experimenter-assigned parameters determined during pilot trials, and the vector 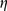 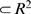 is a noise term taken from a pseudo-random uniform distribution. The vectors 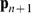 and 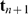 are calculated while frame 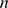 is on the screen and then projected simultaneously in order to create frame 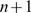. This means that the movement of the pointer figure and reaction of the target figure to it always lag a few milliseconds behind the actual mouse movements. This, however, seems to be an unnoticeable lag. Furthermore, it is close to the ranges of regular computer operation and, hence, does not constitute a departure from the kind of tool we expect our participants to be familiar with.

The participant controls the pointer via the mouse and tries to keep the target within a small gray area in the center of the pen. In order to reduce the likelihood of exponential divergence in terms of the target disappearing from the screen, its escape away from the pointer is scaled down by the parameter 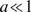. Additionally, the noise term in the target behavior prevents the participant from approaching and trapping the target object under the mouse pointer and, thus, establishing asymptotic convergence to a steady state.

Establishing these constraints makes the task strikingly resemble pole-balancing. We purposefully fashioned the current task as a simulation of balancing a pole on a finger where the target object stands for the pole's center of mass projected onto the hand's plane and the pointer object stands for the point of contact between pole and finger. The more the pole tilts, the more its center of mass goes away from the point of contact and eventually the pole falls down. Meanwhile, establishing completely stable control is impossible, and one has to sway her finger along with the pole. In such a study Treffner and Kelso [35] suggest that the configuration of the task near such critical instabilities allows the behavior to unfold across multiple spatial and/or temporal scales. This gives our approach even more validity since the authors utilized similar time series analysis techniques to confirm 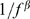 type noise.

During the initial period the mouse performs as a properly functioning mouse would. Beginning at a point in time randomly chosen from the interval 30 plus or minus six seconds after trial onset, a 3-second-long perturbation in the relation between the mouse and the pointer on the screen instantiates a malfunctioning mouse. The pointer dot is not directly mapped from the mouse coordinates to the screen. Instead, an “error” dot location was assigned according to the following expression,
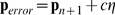
(2)where 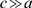, 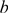 is an experimenter-assigned parameter, and the vector 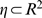 is a noise term taken from a pseudo-random uniform distribution. The result is that for three seconds the pointer object jumps around its “proper” location on the screen as assigned by the mouse and consequently it becomes hard to control. (See Figure 1.)

OptoTrack motion-tracking apparatus (Northern Digital Inc., Waterloo, Ontario, Canada) using a separate computer stored the three-dimensional coordinates of a hand marker at a sampling rate of 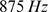. We used a velcro band taped around the proximal phalanx of the ring-finger to secure the infrared marker. All six participants used the right hand to handle the mouse.

The verbal counting task requires starting from a randomly chosen number between 90 and 100 and counting backwards by three. The participant was told to start over from 100 if she reached zero before the end of the trial. In the second experiment, we simplified and made more reliable the encoding of the cognitive performance dependent variable by setting the perturbation to always begin precisely 30 seconds into the trial and last for six seconds. Additionally, a starting number of 400 was chosen for the counting task such that counting would happen in the range of three-digit numbers. No motion-tracking apparatus was involved and, instead, a digital video camera was placed behind the participant and aimed towards the monitor screen to record the whole session and was used later for the encoding of counting rate.

### Analysis of Motor Behavior

The data in experiment 1 were analyzed using Detrended Fluctuation Analysis (DFA), a technique which allows us to estimate a coefficient of temporal correlation in a time series [36], [37]. Since Mandelbrot [38] proposed to generalize Brownian motion using fractional Brownian motion as including correlated displacements, several related techniques for quantifying the noise (the derivative of the trajectory) have been developed. While the coefficient 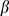 in 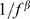, the slope in the logarithmically transformed frequency power spectrum, estimates power-law scaling of activity amplitude in the frequency domain, the 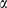 exponent from DFA estimates power-law scaling of variability in the time domain, and both 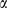 and 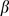 quantify temporal correlations in the time series [37]. We chose to use 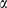 here instead of 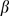 because, first, these two coefficients are related as 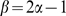 [19], [39], and second, DFA has some advantages over other methods with respect to its robustness to non-stationarities in the data [40]. In the context of behavioral measurements, DFA has been applied to a wide range of studies, including heartbeat [41], walking [42], postural sway [43], tapping to a repetitive signal [22], and EEG recordings [44].

The analysis begins with transforming the data into the appropriate variable. The raw data was filtered using a 60-Hz low-pass filter and down-sampled by a factor of 10. Only lateral displacement of the mouse in the horizontal plane of the table was used because a one-dimensional time series is sufficient for applying DFA and similar techniques. The filtered lateral position data was differenced twice to obtain the acceleration. The acceleration series is the relevant variable here since it corresponds to the active control on the part of the participant, i.e., the participant's active changes of the movement of the mouse. In order to make a correction in the trajectory of the mouse related to inducing a correction in the trajectory of the target, the participant needs to apply force on the mouse, that is, change its acceleration.

**Figure 2:**
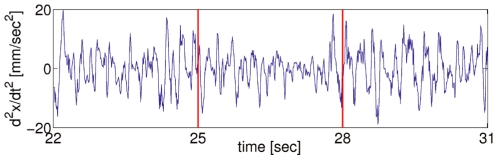
A portion of the analyzed acceleration data from a representative trial. The section in the middle delineated with vertical bars was collected while the mouse was malfunctioning.

The input data (see Figure 2) with length 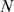 is integrated to form the cumulated sum or profile. Then, a series of fluctuation functions 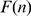, the RMS of the residuals of a linear regression fit inside each block of a sequence of all consecutive blocks with size n, is calculated per size 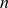 of the observation window according to the following equation
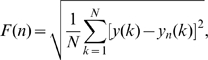
(3)where 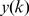 is the integrated time series within a block and 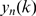 is the linear trend in the same block.

In a step-by-step fashion, the algorithm can be described as follows. The first block 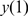 with length of 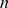 samples is taken from the integrated time series and the linear trend inside it, calculated by fitting a first-order polynomial, is subtracted from it. The operation is repeated for all consecutive non-overlapping blocks of data with length 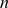. This is the detrending step which produces a series of residuals with length 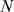. Finally, all terms in the residuals series are squared and the root is taken from their mean to obtain 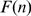. Next, the same procedure is repeated using a different value for the binning parameter 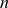.

**Figure 3:**
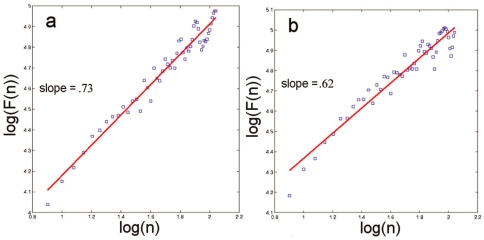
Fluctuation functions from representative five-second blocks covering behavior with proper (a) and malfunctioning (b) mouse.

Naturally, 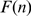 increases as 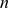 increases but more importantly, in case of a linear relationship between them in a log-log plot as in Figure 3, the exponent in the power-law relation 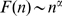 corresponds to the scaling parameter and has been used to identify persistent or positively correlated process when 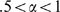 [42]. In the special case when 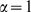 it is equivalent to ideal pink 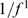 noise. Thus, finding values of 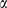 significantly higher than .5 indicates the presence of 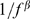 type noise, and hence a smoothly operating IDS.

**Figure 4:**
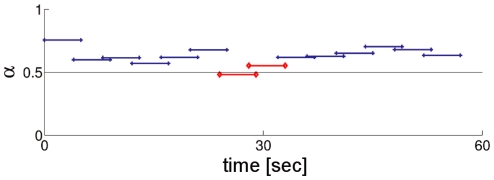
DFA results per block for a representative trial. The x-component of the lines stands for the time coordinates of the analyzed block and the y-component is the scaling coefficient obtained for that particular block. The red lines in the middle are the ones that cover the perturbation section of the trial.

In order to quantify the evolution of the temporal correlations in the hand-tool displacements during the whole trial, we applied DFA recursively to 5-second-long blocks with increments of 4 seconds. Figure 4 portrays a series of 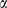-coefficients for a given trial. In order to evaluate the effect of mouse perturbation, the coefficients were divided into three groups and averaged to obtain mean coefficients for the blocks covering the time series before, during, and after the perturbation.

### Analysis of Cognitive Performance

Using the video recording we encoded the number of times a number was pronounced during each of 10 consecutive 6-second-long blocks within a trial. Consequently, three average counting rates per participant for the periods before, during, and after perturbation were obtained by dividing the total number of counts in the blocks covering a period by the respective number of blocks. For example, the average pre-perturbation counting rate is the number of counts for the first five blocks divided by five.

## Results

### Scaling Coefficient 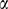


**Figure 5:**
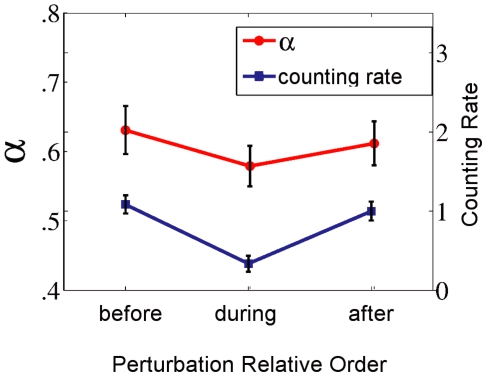
Means of the main measures used in the two experiments. Counting rates are averaged across consecutive 6-second-long blocks. Error bars are standard errors.

First, we evaluated the hypothesis that hand-tool displacements analyzed for each of the three groups of averaged 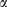-exponents exhibited 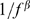 type positively correlated noise diverging from the 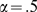 level characteristic of white noise. Separate 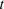-tests indicated that the pre-perturbation (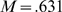, 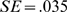) and post-perturbation (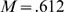, 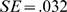) ones diverged, 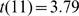, 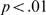 and 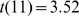, 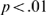. The perturbation group, (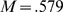, 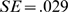), albeit barely, also made it to the level of a significant difference, 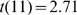, 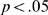, with a lower confidence interval boundary at 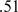. Repeated measures ANOVA (RMANOVA) with perturbation-relative order as independent variable and averaged 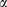 coefficients as dependent variable, illustrated in Figure 5, indicated a significant effect of perturbation, 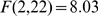, 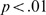. Accordingly, the mouse perturbation reduced the long-range correlation in hand-mouse displacements relative to the ones in pre-perturbation (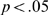) or post-perturbation (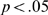) periods. The latter two did not differ (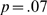).

### Counting Rate

Four participants had to be excluded from the analysis of Experiment 2 because two failed to perform the motor task accurately, that is, they allowed the target to leave the playground area, and two others forgot to restart the additional cognitive task after the perturbation. For the remaining nine (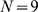), consistent with the motor behavior results in Experiment 1, RMANOVA with perturbation-relative order as independent variable revealed a significant effect of perturbation on counting rate, 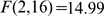, 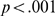. Illustrated in Figure 5, pairwise-comparisons revealed that the average counting rates for pre-perturbation (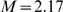, 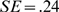) and post-perturbation (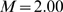, 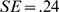) did not differ (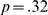) but were both significantly different (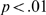) from counting rate during perturbation (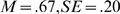).

## Discussion

### Noise at the Body-Tool Boundary

**Figure 6:**
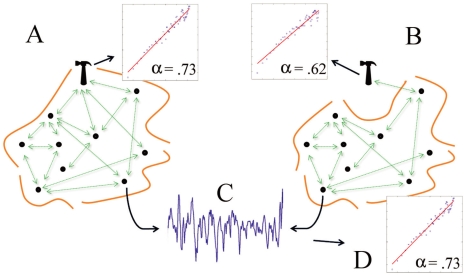
A schematic of two distinct model agent IDSs. The IDSs (delineated by the surrounding curves) are fluidly or softly assembled by virtue of rich interactions on multiple scales (double-sided arrows) among the components (black dots and hammer) are portrayed. They either span across (A) or do not (B) the tool (hammer). It is assumed that the black dots stand for bodily structures. Notice that interaction between the tool and the agent is present in both cases but in (B) it is impoverished, i.e. constrained to a single scale. Customarily, one studies such systems by collecting a time series locally from the behavior of a single point of observation (C), that is, from a single element. Next, if possible one establishes their character as an IDS by searching for power-law scaling of certain statistical quantities (D) as it was done, for example, in [21], [25], [26]. The two fluctuation function plots representative of our data exemplify analysis of the behavior of the tool in (A) and (B) and were obtained using DFA which we applied instead of power spectrum analysis. The scaling coefficient 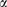 reveals long-range correlations characteristic of 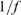 noise in the hand-tool in normal mode (A) and approaches the uncorrelated white-noise level in (B).

In Experiment 1 we find evidence to support our prediction that during skilled task performance the behavior of the hand-tool will exhibit the kind of power-law scaling associated with 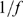 noise [18]. Consequently, although one can distinguish anatomically between separate behaving components, i.e., parts of the tool, body segments, neural pathways, etc., the task performance is more appropriately understood by taking the tool to be functionally integrated into a larger IDS, the body-tool IDS. As expected, a sudden alteration of the connectivity between certain components disrupted task performance generally. Since dynamics play a constitutive role in a softly-assembled perception-action device [21], [45], their disturbance leads to disruption of the integrity of the device. This disruption is manifest in the significant decrease of the scaling coefficient characterizing long-range correlations, or “whitening” of the noise while the mouse misbehaves. Notice that while the anatomical relation between the mouse (the tool handle) and body has not changed, functionally they act much more like separate components interacting on a restricted local scale as portrayed by Figure 6 a and b. That is, the mouse switches from being an intrinsic part of a self-assembled device solving a certain problem to the problem that a newly assembled IDS has to solve.

While we aimed for an obvious categorical distinction between tool dynamics with and without perturbation, we observed only a drop in the scaling coefficient without a complete transition to a behavior characterized by white noise. Yet, this is not necessarily a weakness in our argument. Remember that Heidegger never establishes in a precise manner the nature of the unreadiness-to-hand as a special mode of being of equipment. Instead, it is placed in a gray area between presence-at-hand and readiness-to-hand and is usually regarded as a deficient, still a subordinate mode of the latter [10], [11]. This conceptualization matches our experiment. During pilot trials we tuned the magnitude of the perturbation so that is does not give rise to a complete break-down; the participant was capable of maintaining a certain level of control throughout. During experimentation only two participants proved incapable of performing the task with the perturbation and allowed the target figure to escape from the pointer figure by leaving the screen. Hence, it makes sense to say that, just as it is in Heidegger's example, we instantiated a situation where the tool misbehaves but retains some of its “usability”. Consequently, our reliance on dynamics at the tool-hand interface to parallel the phenomenological description of the two modes - readiness-to-hand and unreadiness-to-hand - is justified. We observed a certain level of long-range correlations with both a properly and improperly behaving tool, but the exponent was decreased significantly in the latter case.

Another important clarification is needed here. Information about the mouse never stops being available to the participant, even during a period of perturbation. This is a crucial point since for an object to be phenomenologically unready-to-hand, the user needs to be interacting with it. We are not arguing that the flow of interaction between tool and body is reduced in magnitude, just that it is reduced in complexity. The mouse keeps providing sufficient local stimulation through the eyes and the sense-organs of the arm for the agent to maintain overall control over it, as when one is holding a foreign object in hand and is trying to figure out a specific property of it.

### Cognitive Load

In accordance with our hypothesis, Experiment 2 revealed that the effect of the motor perturbation apparent in the significant drop in counting rate is not localized to the body periphery but extends to other levels of behavior involved with the task. This satisfies the second criterion for our operationalization of Hedeigger's proposed transition from readiness-to-hand to unreadiness-to-hand. Before the perturbation, the tool, by way of the mouse, its handle, is functionally a component of the smoothly coping IDS. Experiment 1 showed that this is less so during the perturbation. If this disruption of smooth coping constitutes also a shift from readiness-to-hand to unreadiness-to-hand, the mouse handling will start to emerge as another object of attention. This would increase the participants' cognitive load, and lead to decreased performance in some of the ongoing cognitive tasks.

Because experiment 2 tracks attention only indirectly, other explanations of the decrease in counting rate are possible. We believe that the attentional shifts due to increased cognitive load is the most plausible explanation of the decreased counting rate, especially considering the simplicity of the experimental task. In support of this interpretation of the results, it is worth noting that our reason for excluding two participants from our data analysis is that they completely discontinued the counting task during perturbation and, furthermore, failed to restart counting after the perturbation. We take this is as indicating that, for these participants, the perturbation caused such a profound shift in attention to the herding task that the counting task was excluded entirely.

It is important that participants could easily bring performance back to its regular state of affairs. Probably it takes some time to recover from such an environmentally-induced attentional strain. The average post-perturbation counting rate and 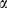, however, were not significantly lower than the respective pre-perturbation values. This allows us to maintain that we have correctly created a setup involving the mouse, screen, and specific game that is not too taxing for our participants even during perturbation and allows them to use a tool they are fairly familiar with, something that is another line of convergence with Heidegger's description of ready-to-hand.

One could ask how extreme a perturbation one can induce. According to Eq. 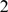, the pointer behaves erratically, but it still follows the mouse roughly. During pilot trials we tuned the parameters 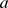, 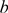, and 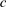 such that the task became challenging but not impossible even at its hardest stage. In this way we created the conditions for the tool to become obtrusive and to require some attention without interrupting the experimental tasks. This comes closest to Heidegger's notion of unready-to-hand. But one can extrapolate from the current study and imagine the extreme case of total tool breakdown when the pointer stops responding to the mouse at all. Then, the motor behavior would most likely be discontinued and the participants' attention would be diverted completely away from herding and counting. This would be an example of presence-at-hand, Heidegger's third mode of being for equipment.

### General Discussion

As noted in the introduction, Heidegger's phenomenology has been influential in the cognitive sciences, despite the fact that no attempts have been made to empirically confirm his insights. The experiments in this paper support Heidegger's description of the transition from readiness-to-hand to unreadiness-to-hand, a phenomenon that is key for his overall phenomenological philosophy. When humans are smoothly coping with entities ready-to-hand, they see through their tools to focus on the task they are using those tools to complete. When that coping is disrupted by a temporary malfunction, humans can no longer see through the malfunctioning tool and experience it as unready-to-hand. We demonstrated this transition by showing that when participants smoothly operate a mouse in a video game task, the body-tool performance displays the complex dynamics typical of an IDS. Temporarily disrupting mouse behavior temporarily disrupted this IDS, at least at the body-tool boundary. We also showed that this disruption led to a reconfiguration of the participants' awareness of the situation by showing a shift in resources allocated to an additional cognitive task. This is closing in on Heidegger's transition from readiness-to-hand to unreadiness-to-hand. We take these experiments as progress toward justifying the influence that Heidegger's phenomenological philosophy has had on cognitive sciences and justifying the partly Heidegger-inspired claim that cognitive systems sometimes extend beyond the biological body.

A major challenge in using experimental work to validate phenomenological observations is that these might seem to be two orthogonal planes. This is why we needed to check our predictions related to extended cognition as derived from Heidegger against predictions derived from an additional theoretical approach to perception-action [21], [25], [26]. More interestingly, in the context of the current study these two perspectives enriched each other's predictions. Without the notion of a self-assembled device with interaction-dominant dynamics resulting in 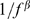 noise, it would be hard to predict what kind of change of motor behavior would result from a perturbation of smooth coping. Similarly, until one considers Heidegger's discussion of ready- and unready-to-hand, speaking of an IDS as incorporating or failing to incorporate a tool does not necessarily inform you of the size and direction of the impact on awareness that a functional perturbation of that tool is going to have. The combination of the two approaches provides for a model that explains the observed data.

The experiments described above have two further implications. First, Experiment 1 lends support to an untested hypothesis found in [21]. Particularly, the authors claim that 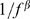 noise characteristic of cognitive behavior should be observable even in relatively fast scales of motor activity at the periphery of the body, and not just in tasks the responsibility for which is traditionally attributed solely to the central nervous system. Second, the experiments provide further evidence in favor of the hypothesis of extended cognition. That is, our demonstration of the presence of 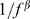 long memory process during smooth coping and its reduction during perturbation of that smooth coping supports the notion that the body-tool instantiates an IDS.
